# Mapping power in community engagement: methodological reflections from health emergencies

**DOI:** 10.1186/s12874-026-02905-1

**Published:** 2026-06-17

**Authors:** Luisa Enria, Giulia Loffreda, Angus F. Tengbeh, Abass S. Kamara, Alie D. Timbo, Karin Diaconu, Sophie Witter

**Affiliations:** 1 Department of Global Health & Development, London School of Hygiene & Tropical Medicine, London, UK; 2 Rebuild for Resilience, Institute for Global Health and Development Division, Queen Margaret University, Edinburgh, UK; 3 Alliance for Health Policy and Systems Research, World Health Organization, Geneva, Switzerland; 4 Ministry of Health, Freetown, Sierra Leone; 5 College of Medicine and Allied Health Sciences (COMAHS), Freetown, Sierra Leone

**Keywords:** Community engagement, Health emergency, Qualitative research, Participatory methods, Sierra Leone

## Abstract

Community engagement is increasingly seen as a key component of public health responses to health emergencies. Recent outbreaks, from Ebola in West Africa to COVID-19 across the world, have highlighted the importance of working through existing structures and identifying trusted local leaders who can help response workers design context-appropriate interventions. However, community engagement efforts often rely on homogenous understandings of ‘community’ that underestimate the significance of power. This creates a risk of exclusionary practices. In this article, we discuss the use of participatory power mapping workshops as a useful method to identify who has authority and who is trusted by whom, and consider its implications for how we think about trust-building during emergencies. We detail the implementation of this method using participatory workshops conducted in Sierra Leone. We show how these workshops provided granular insights on authority structures and the relationship between power and trust. These discussions highlighted that trust is not homogeneous nor static, and helped kickstart conversations about the role of citizens not simply as recipients of health interventions but also as active participants in how health interventions are designed and delivered. Tracing power dynamics with and within communities can help develop allows us to make power visible in health interventions, maintaining a critical lens on how interventions themselves intervene dynamically on its distribution. In practice, a systematic application of this methodology can encourage more diversified community engagement efforts, and potentially set the foundations for citizen-led collective action around health emergency resilience.

## Introduction

Community engagement is increasingly seen as a key component of public health responses to health emergencies. Defined by the World Health Organisation as a “process of developing relationships and structures that engage communities as equal partners in the creation of emergency response solutions that are acceptable and workable for those they impact”, community engagement is expected to “empower communities to confidently share the leadership, planning and implementation throughout the health emergency cycle” [1]. The formal inclusion of community engagement in health emergency response is a relatively recent phenomenon, as the first social mobilisation and community engagement “pillar” was introduced alongside more traditional pillars (e.g. case management and logistics) during the 2014–16 West African Ebola outbreak [2]. As several scholars, as well as official review panels assessing the effectiveness of the Ebola response, have noted, in the first months of the Ebola virus spreading across Liberia, Guinea and Sierra Leone, community experiences and structures were not effectively taken into account [3–5]. The epidemic was treated solely under the purview of biomedical experts, with the support of the security sector as across the three countries, foreign and local militaries were brought in to enforce quarantines and curfews as well as providing logistical expertise [6]. Over time, it became clear that this approach was not working. As social scientists working in all aspects of emergency operations highlighted, communities’ trust was essential for the effective implementation of public health measures, and that to achieve it, emergency responses needed to be tailored to local realities [7, 8]. Not only that, but people had begun implementing their solutions, and existing community structures and leadership were recognised as playing a key role in helping build confidence and ownership in the response [9]. Community engagement efforts, such as the Social Mobilisation Action Consortium (SMAC) in Sierra Leone, had a demonstrable impact on the course that the epidemic took—increasing, for example, the number of reported safe burials, amongst other indicators [10]. Lessons from Ebola have been applied to subsequent epidemics, and the sector has become increasingly formalised through Risk Communication and Community Engagement (RCCE) pillars [11].

These experiences have highlighted the importance of working through existing structures and identifying trusted local leaders who can help response workers design context-appropriate interventions. This has been deemed important not only for acute crises but also to develop community-centred approaches to health emergency preparedness [12]. Indeed, across all health research and practice, patient- and community-centred approaches, including growing emphasis on public and patient engagement, are increasingly expected, including by funders and major health agencies. Whilst the recognition for the essential role of localised responses and community-centred practice has been widely welcomed, there remain important critiques surrounding the often ‘uncritical’ use of the term ‘community’ when it comes to conceptualising community engagement for health emergency and preparedness programming. This refers, for example, to the risk that ‘community engagement’ assumes the existence of a visible and often homogenous ‘community’ that can be meaningfully engaged as a unit. In contrast, Wilkinson et al*.* note that, in practice, “communities and their boundaries are inherently subjective” and that “simplified notions of community, which gloss over differences, divisions and multiple identities within particular locales” may ultimately “undermine the best intentions to include local people in the design process” [13]. They propose that the “lesson from Ebola…is not that ‘communities’ can stop epidemics and build trust, it is that understanding social dynamics is essential to designing robust interventions”. Similarly, there can be significant variation in what engagement means and how it is practiced— ranging from information campaigns to deep involvement and ownership.

Key to understanding social dynamics for designing interventions is an appreciation of how power operates within communities. Community engagement efforts that rely on conceptions of ‘community’ as homogenous may for example make simplistic assumptions of who is a ‘community leader’, failing to pay attention to internal divisions and contestations of formal authority structures. For example, in ethnographic research on the Ebola vaccine trials, researchers found that whilst the use of chiefs for social mobilisation was met with outward community support, divisions and contestations were to be found behind closed doors [14]. For some members of the community, perceptions that chiefs had profited from the epidemic led to mistrust, and thus required a more diversified engagement strategy. Qualitative research therefore has a key role to play to help develop a granular understanding of who has authority to speak for whom, whose authority is trusted and whose is contested (and by whom) and who is able to bring about or resist change. Asking this question can help identify salient divisions and social groupings and understand which groups and individuals are viewed as legitimate and trustworthy and which forms of power are instead challenged. Such an analysis brings power back in view, challenging simplistic notions of community that hide more than they reveal. In practice, this allows for the development of engagement strategies that can be tailored to different groups, and which can adapt as social dynamics change over time.

In this paper we present a participatory methodological approach to explore power dynamics. Power mapping emerges from a long-standing tradition of participatory research, originally associated with Participatory Rural Appraisal (PRA), and it has been primarily applied to studying development and natural resource management. Different researchers use different tools to map power, but all of them are intended to “provide a visualisation of local actors and the group of social forces” that help explain how power operates in particular localities [15]. Here we put forward our adaptation of power mapping tools for application by global health researchers and practitioners, with a particular focus on how they may be used to study power for health emergencies response and preparedness. The adaptation of these tools for mapping power in health emergencies is particularly important precisely because of the analytical and operational urgency of understanding social dynamics in crisis contexts. In particular, we point to the crucial role that this adaptation of power mapping methodologies can play in disentangling the relationship between power and trust, for example challenging assumptions that one entails the other. Being able to understand which forms of formal and informal public authority are trusted or otherwise, and therefore who has legitimacy to support health emergency interventions, can make or break a crisis response.

We first discuss the methodology in detail, then reflect on its implementation through two case studies in Sierra Leone to help us think through what the implications of understanding power might be in the fields of preparedness, risk resilience and beyond.

### Understanding power

In the social sciences, the concept of ‘power’ has been widely theorised as well as contested. Although there is an overall consensus that “power usually refers to control, authority or domination, which involves the ability to influence the decisions and behaviours of others” [16] much has been written about *how* and *by whom* such power is exercised. Over the course of the twentieth century, for example, social theory moved beyond the association of power simply with control over the means of force by the state to observe the more diverse and diffuse ways through which different individuals and groups of people exercise influence. Marxist theorists such as Antonio Gramsci, for example, referred to ‘hegemony’ as a way of pointing to how ideology “imposes or reinforces (through conditional consent) a certain social order” [16]. Similarly, Michel Foucault further highlighted the intimate connection between power and knowledge and the subtle ways in which institutions such as schools and prisons have deep implications for citizens’ discipline and self-discipline [17, 18]. These theorisations alert us to the fact that researchers should look for power not only amongst those with formal positions of authority but much more broadly within society. In addition, when it comes to engaging communities in the design and implementation of public health and health research, we are not solely interested in who has the ability to exercise control but also in who is trusted, or who is seen to have legitimacy when it comes to influencing the decisions of others. For this, the concept of “public authority” is useful as it has been applied by researchers at the Centre for Public Authority and International Development (CPAID) in relation to health emergencies [19]. Their research shows the significance of a wide range of sources of authority for how people across contexts experience and engage with health emergencies. Such authority can be “any kind of authority beyond the immediate family which commands a degree of consent”, and as such it may have “little to do with state institutions”, with legitimacy placed instead in faith-based groups, kinship networks, civil society groups or others [20, 21]. These groups may influence, for example, what health information and interventions are trusted, the uptake of different types of public health measures (e.g. vaccination) and the possibility for co-design and collaboration between public health workers and those affected by health emergencies. In contrast, attention to “public authority” can also make visible how certain forms of control may be “unaccountable” and serve to enforce authoritarian forms of rule, as was the case in Parker et al*.*'s research on the military’s enforcement of COVID-19 control in Uganda [22]. Similarly, paying attention to different forms of public authority can cast light on how perceptions of different forms of power may shape citizens’ interpretations of health emergencies, as evidenced by fears during both Ebola and COVID-19 epidemics that vaccines were being used by governments to decimate their population, highlighting high levels of mistrust in state institutions amongst certain constituencies [23]. In addition, it is important to recognise that authority is “unsettled”, as it is subject to constant redefinition [24]. This should also include an appreciation of how public health interventions themselves reshape distribution of power within communities. A close analysis of power in efforts to intervene in and prevent health emergencies also requires an understanding of how public health responses themselves reshape distributions of power within communities. In Sierra Leone’s borderlands, for example, our research notes how narratives around community engagement in the Ebola response portrayed authority as if it was something historically fixed, out there to be discovered. In contrast, our showed how research showed how becoming gatekeepers of the Ebola response emboldened the power of paramount chiefs, whose role had been challenged during Sierra Leone’s civil war, and then again how tensions following the Ebola response led to renewed disputes over their authority. Legitimacy, in other words, is not given but should rather be treated as “an object of study, which develops and changes, including as a result of encounters with humanitarian interventions” [24]. Building on these theoretical foundations, power mapping methodologies can help re-centre power in global health research and practice, making its workings visible, allowing us to trace its distribution.

### Mapping power in practice

In this paper we reflect on how we have used power mapping methods across different research projects in three areas of Sierra Leone: Moyamba, Kambia and Kailahun Districts. In Kambia, we used participatory power mapping to explore social dynamics relevant to epidemic preparedness, including the rolling out of experimental, emergency and routine vaccination, particularly in villages on the border with Guinea that had recently experienced Ebola and measles outbreaks. We used power mapping approaches in two main ways: workshops facilitated by social science researchers and workshops facilitated by Community Health Workers (CHWs) who had been trained in citizen ethnography, that is, the training of lay people in basic ethnographic methods and data analysis. The CHWs conducted 5 power mapping workshops, informed by daily ethnographic observations over a period of 2.5 months. In Moyamba and Kailahun, we used this method to explore ways to engage communities and their leaders and incorporate their views into local decision-making related to health and public health interventions; the aim was to promote community health system resilience, particularly during shocks and crises. Here we conducted 6 workshops (3 per setting) over the course of a week. Researchers who were already familiar with the method facilitated the workshops, which focused on mapping the varied stakeholders that were influential in responding to any type of health shock or local health need. Additional learnings for this paper are drawn from the work done in Kambia for a number of different projects, including one on the political economy of vaccine deployment for which two power mapping workshops were conducted at Peripheral Health Units as well as ethnographic research on power for a project on the politics of knowledge and (mis)trust in epidemic preparedness and response conducted over seven months.

Across sites, power mapping was used as a tool amongst a broader programme of research. This allowed us to use the power maps to stimulate discussion about power and about social dynamics within each locale, and to use them as a starting point to trace the relationships highlighted in the workshops over time and in more depth.

We present first the overall methodology and then some reflections based on our experience of conducting these workshops. The approach to power mapping for these projects were adapted from the one developed by Christian Aid [25]. This tool refers to power mapping as a form of stakeholder analysis used to understand “who, in a given context, has what ability to create or resist change”. Participants for each workshop were chosen through purposive sampling to reflect a broad representation of the community. In each location, these were selected between research collaborators and researchers with deep local knowledge, following research within the community and in conversation with district health authorities. This selection had to be done carefully as, as discussed in more detail below, there is a danger that power dynamics can be exercised through the selection of participants and during the workshop itself, with some people feeling uncomfortable expressing their views if they feel that people in positions of influence are present.

The workshops were conducted following five key steps:

- Step 1: Defining power

Participants are asked to discuss what power means to them, with a facilitator steering the discussion around the particular topic of interest—e.g., who has the ability to create or resist change around, for example, vaccination uptake. In this discussion, participants are also invited to consider what different kinds of power exist and what makes different people powerful, e.g., knowledge, resources, or positions. The facilitator here also emphasises that power need not be formal, and that it can be more or less visible, so as to ensure that participants do not simply list people who have official authority in their community.

- Step 2: Mapping power

Participants are invited to list people in their community who they see as powerful in relation to vaccine uptake, health emergency resilience (or any other specific topic). The facilitator asks participants to discuss among themselves and tease out any disagreements. Once participants have listed all stakeholders, the facilitator asks them whether any are missing or perhaps if they identify some people that have not been named; they may ask why they are not part of the list to elicit any further insights into the workings of power within each local area.

- Step 3: Identifying levels of power

Looking at the map produced in the previous step, participants are then asked to determine the relative power of different stakeholders (e.g. by drawing smaller or bigger circles around individual names).

- Step 4: Identifying trusted stakeholders

Participants are then asked to look and reflect on the listed stakeholders to differentiate between powerful people who are more or less trusted. Once participants have listed a stakeholder, the facilitator asks participants to discuss amongst themselves whether the stakeholder is more or less trusted, teasing any disagreements.

- Step 5: Identifying mechanisms of power

Finally, participants are asked to discuss what makes different individuals powerful—e.g. using different colours to denote whose power is derived from a formal authority, their knowledge, their resources, their disposition, or any other factor that the group deem relevant.

- Step 6: Interpretation

Finally, once the map is complete, the group is asked to look at it and discuss what we can learn from the map and identify any surprising findings. The map is then used to discuss what this means for how we might go about bringing change—e.g., for vaccine uptake or emergency preparedness.

The power maps are the main output of the workshops, as they produce a visual representation of participants’ perspectives on how power operates within their communities in relation to the chosen topic of discussion. For our case studies, the workshops were also recorded and transcribed so as to be able to analyse discussions and debates during the mapping process to tease out specific reasonings for assigning more or less power, attributing trust, and deciding different mechanisms and dynamics of power. These transcripts can be analysed through a thematic approach as for other types of Focus Group Discussions. In our projects we coded the transcripts manually, but data analysis software such as NVIVO can be used. In Kambia, for the citizen ethnography work with Community Health Workers, we also held a collaborative analysis workshop between the CHWs and the District Health Management Team to reflect on key findings and to input into the redraft of the District’s community engagement strategy for vaccination during and after health emergencies. In Kailahun and Moyamba, after each workshop, we presented back to participants what emerged from the previous discussions to ensure agreement on the final map.

The research discussed here was given ethical approval by University of Bath (where LE was originally based), the London School of Hygiene & Tropical Medicine, Queen Margaret University and the Sierra Leone Scientific and Ethics Review Committee. In both studies, research teams comprised of a team of social scientists from UK and Sierra Leone-based universities, as well as including the active participation both as researchers and as analysts of local health workers, including public health officials and Community Health Workers. All participants in the workshops were provided with information sheets and consent forms. We have written elsewhere about specific questions surrounding positionality and reflexivity that emerge when asking CHWs to conduct research in their own communities [26].

### Key observations from running power mapping workshops

Our power mapping workshops across the study sites yielded results that, despite contextual variations, pointed to important common observations. To begin with, formal positions mattered. Across all three sites, we found that positions such as paramount chief or elected local officials were deemed to be powerful. Similarly, in Kailahun in particular, the District Health Management Team was seen as a powerful player. Secondly, trust was not static or homogenous. For the community resilience workshops, for example, participants were divided into three different groups—community leaders, informal leaders and community members. Even in the same locality, these different groups identified different individuals as being trusted and powerful. Similarly, types of actors that may be seen as trusted and powerful in one place may be less significant in others. This was the case for NGOs, which were identified as powerful in all workshops in Moyamba and only one in Kailahun. In Kambia, discussions tended to focus on actors within the community, although the complementing ethnography pointed to the significance of groups such as the DHMT and NGOs in shaping community dynamics. Across most workshops, religious leaders were identified as a powerful and trusted group. In one workshop in Kambia, for example, the imam was identified as being powerful and trusted by virtue of his knowledge. Interestingly however, he was not, in this particular community, seen to be as powerful as the local youth leader whose social standing came from his social skills and ability to relate to others in his community, highlighting the range of attributes that contribute to who is perceived to be powerful. In Kailahun and Moyamba, religious leaders were described as having the ability to shape public opinion and mobilise support, address social issues, with their roles extending to advocacy, peacebuilding, and interfaith dialogue. In these discussions, traditional healers held an ambiguous role—being recognised for example as important actors, particularly in areas with limited access to formal healthcare, but then often designated as not being as powerful or trusted as other leaders. In one workshop in Kailahun with participants including members of the bike riders’ association, religious leaders and market women, for example, healers were identified as powerful, because they can cure illnesses in the community or because their looks can make them fearsome. However, when asked to rank them in relation to other actors, participants settled on a lower score out of ten with the following collective deliberation:

- *R1 They should be ranked at 3… They can be very opposed to health emergency laws… They do not uphold laws governing disease outbreak… they can create so much problem by spreading infection…*
- *R4: They can be very lawless… So, I will rank them at 1*
- *R6: Their actions can be deadly when they defy the government by refusing to refer patients to health facilities…They can keep patients until it’s too late for the formal health system to do anything about it…So for that reason, let’s rank them 3…*
- *R2: Even though they can be lawless, they are still vital in society… Their lawlessness were more visible during the Ebola outbreak but thing got better with COVID-19… They were somewhat compliance and have been regular in health emergency meetings even more than the BRU and market women… So, let’s rank them at 3…*

This may also be the result of the stigma often associated with traditional medicine. Other traditional leaders such as *soweis* (female secret society heads) were mentioned in some workshops as having influence particularly amongst their members.

Crucially, deliberation also at times surfaced disagreements and efforts were then made to arrive at a collective conclusion although it was not always possible to reconcile divergent opinions. An example of this, also from the Kailahun workshop when it came to discussing the role of the bike drivers’ union:

- *R2: I would rank them 5… They can be active in certain areas, but not so good in other areas… They can have both positive and negative influence in communities.*
- *R1: I think they should not be ranked above 5… Their compliance with health regulations especially during outbreaks is not good…*
- *R4: They most often disobey law and order by plying with more than required passengers… Overloading is a big issue among them, which can cause diseases to spread quickly… So let’s rank them at 2*
- *R6: I think we should still leave them at 5… They can have their bad sides but can also play significant roles in transporting sick people to health facilities where vehicles cannot access at that time of need.*
- *R3: I will go with 5… they can willingly go to hard to reach areas where vehicles are most often reluctant to go. They play critical role in that sense… They can also be take the position ambulances when one is unavailable by saving lives…Even CHWs are sometime recommended to be moving with one bike rider to one CHW… So let’s rank them at 5…*

In this instance, the moderator decided to go with a ranking of 5 as the majority response.

A focus on categories of actors however may obscure what was another key finding across our workshops—namely that not all who are powerful have a formal position or recognised role in the community. In several workshops in Kambia, for example, participants identified individuals by name, stating for example “Aminata^1^ is also highly listened to, she does not have any formal powers, but she is very instrumental”. In another village in Kambia, one man was identified as powerful because he had been “selected by our community to be a journalist: he champions issues in our community”. Significantly, whilst power and trust were often associated, this was not always the case. In one village in Kambia, for example, a chairlady was assigned ‘medium’ power by virtue of her position—the same level as the imam, for example—but then, in contrast to the imam, was designated as ‘not trusted’.

The power maps also had important ‘afterlives’, as we traced how the insights from the workshops were used by participants, facilitators and health workers in the sites where the research took place and beyond. In Kambia, for example, amongst the Community Health Workers who conducted the mapping workshops alongside other ethnographic research, these discussions prompted reflections around their approach to engaging communities during vaccination campaigns and outbreak response. In particular, they recognised that they may have missed key people, whose involvement may strengthen citizens’ engagement with public health programming. In our collaborative analysis workshop with the Kambia DHMT this resulted in the redesign of their community engagement strategy to include pre-engagement power mapping exercises to ensure that the right people are involved in the subsequent activities.

## Discussion

In this paper we have focused on methodological insights to highlight how power mapping can be used to explore the dynamics of power across sites and health issues, including to explore health emergency preparedness and resilience. Indeed, the key takeaways from power mapping workshops in three Sierra Leonean communities are not just the specific findings themselves but rather the overarching insight that qualitative research can play a key role in making power visible where much health programming renders it invisible through appeals to ‘community’. Opening up the box of ‘community’ through power mapping can offer a more granular picture of who is powerful and trusted, moving beyond homogenising narratives and challenging approaches that solely target specific categories of ‘community leaders’ to acknowledge the diversity of social dynamics that are specific to each place and change over time. As we have shown, power maps can offer not only visual representations of the distribution of influence and trust in relation to specific health concerns, but also serve as important prompts to start deeper conversations about the lived realities of health emergencies and their legacies, and how local context can shape community engagement and its effects on those it purports to empower. These participatory approaches to understanding social life in the places where health interventions take place can therefore contribute to broader calls for revising the role that citizens play not only as recipients of healthcare but as having a voice in how global health programming and research are designed and delivered. Taking inspiration from research in the context of indigenous knowledge in pollution governance [27], the next step would need to be taking local knowledge and expertise as the starting point, challenging existing paradigms, but also reconsidering who leads discussions on how health emergencies are to be managed.

Turning back to the practicalities of running power mapping workshops, there are some limitations and potential challenges that must be kept in mind when implementing this methodology. The first is that when done through single workshops, power mapping can reinforce rather than challenge static views of power. There is a danger that the ‘map’ itself becomes an object taken to represent a fixed reality about a given place. To counter this risk, it is important to repeat power mapping over time and to complement it with other research methods, such as ethnographic observation, that can offer insights into shifting realities.

Complementing power mapping with other qualitative research methods can also shed light on potential omissions in power mapping workshops. Indeed, mapping exercises do not exist outside of power dynamics themselves, and this may influence how comfortable participants feel in accurately describing their perceptions of public authority in their locales. It can be difficult to determine, for example, whether leaders such as chiefs or imams are named because they do in fact hold power and trust, or whether it is because it would be culturally or politically inappropriate not to say so. Either scenario is an important finding for community engagement practitioners and researchers, as it can for example point to the importance of engaging formal power, whether or not its holder is in fact effective or influential. Conducting longer-term ethnographic observations can help identify these more complex relations, observing power in practice and distinguishing between normative statements and lived experience. Other methods such as social connections mapping can similarly complement power mapping to help tease out diverse aspects of influence and trust [28].

A related point to note is about context specificity and transferability. In this article we have described the application of this methodology in Sierra Leone, which has its own specific political history and structures. The role of the chieftaincy, or that of traditional healers, as well as Sierra Leone’s trajectory from civil war and through an Ebola epidemic, configures power in locally specific ways. These findings emerge from the power mapping exercise, as participants deliberate on who is powerful and who is trusted. Yet, they also require an interpretation that is contextually informed to be able to situate these deliberations in the appropriate socio-political settings. The methodological approach is therefore widely applicable, yet what its application in Sierra Leone told us about power and trust is necessarily contextual.

In addition, the selection of the group to participate in the workshops can determine the levels of comfort participants feel to speak openly. For example, in our workshops we decided to either only have community members without formal positions or to hold different workshops, some only with community leaders and others with people without formal positions. In addition, there may be significant disagreements within the group, which may be addressed by producing different maps or noting the disagreements in the transcripts and notes. Indeed, the discussions held in the group to arrive at an agreed map are as important for analysis as the map itself.

Another reflection relates to whether and how power mapping should be used in practice. In the examples outlined above, we used power mapping largely as a ‘diagnostic’ methodology, to identify current configurations of power in communities and better understand how to work with this over time. As noted, repeated mapping may be appropriate to understand shifting realities, or to enable evaluation of any interventions explicitly aimed at shifting power. Emancipatory and participatory research methods can however also be used to pro-actively reconfigure power dynamics, with power maps viewed as boundary objects to enable this process [29]. Diagnostic or emancipatory approaches are of course not mutually exclusive but there may be different conditions under which one may be more appropriate, or feasible than the other, and being explicit about this with participants. Undoubtedly, the point should not simply be to understand community diversity to better penetrate them with health interventions. Identifying power, both in terms of how it can facilitate or hinder change can serve as a foundation for developing collective action, establishing collective visions for how to challenge current hierarchies or how to leverage social dynamics for transformation. However, it is important to note that this must be done sensitively and always involving communities themselves as co-researchers, rather than as objects or subjects of the research. Indeed, the emancipatory work of reconfiguring power is rarely successful as an externally led exercise and must rather come as the result of collectively decided action within the social groups asking for change.

A final consideration revolves around ethics. Power maps may have individuals’ names on them or other sensitive insights—especially if, for example, the group’s map challenges formal authority. Whilst the specific maps must remain confidential, there are several ways in which their insights can be operationalised. For example, positive endorsements of specific groups and individuals as trustworthy may be communicated to health teams charged with community engagement. When power maps have pointed to individuals or groups who are disruptive or mistrusted, we have across our projects adopted sensitive ways to advise community engagement teams. This may for example be to point to specific areas of the community that may benefit from further dialogue (rather than singling out individuals). Similarly, in cases where formal authority was highlighted as not being trusted, we suggested that although formal authority figures must be consulted, our mapping could point to a wider range of social groups that should also be engaged in dialogue. This is especially important when dealing with sensitive issues such as vaccination or when conducting mapping during or just after a crisis, where vulnerabilities and tensions may be heightened.

In sum, we would caution against the use of power mapping as a way to achieve a definitive understanding of social dynamics, but rather to treat it as a method that can start important conversations about the complexity of authority and trust in a given place and as an invitation to observe its shifting dynamics. When used for community engagement, power mapping’s strength is to identify individuals and social groups that may have otherwise been ignored and to suggest the limits of organising around preconceived notions of ‘community leadership’. Power mapping could be used complementarily to other methods – for example also at more macro-levels in political economy analyses, or even at more micro-levels, as would be the case for social network analyses [30, 31].

## Conclusion

Involving citizens in how we imagine and plan for health emergencies and resilience is crucial to effective and sustainable responses to crises. However, current approaches to community engagement and participation tend to underestimate the significance of power and thus risk becoming exclusionary. Power mapping methodologies, when used in combination with other social scientific methods, can help shed light on who has authority and is trusted and by whom. This, at a minimum, can help bring power into view in critical engagement with global health as well as encouraging more nuanced community engagement approaches. Tools to identify power can however also lay the foundation for citizen-led collective action to take leadership in preparing for and responding to health emergencies.

## Data Availability

Where ethics approvals and participant consent allowed it, data was deposited in the UK Data Service.

## Footnotes

1 Pseudonym.
